# Nano-mechanical measurements of protein-DNA interactions with a silicon nitride pulley

**DOI:** 10.1093/nar/gkv866

**Published:** 2015-09-03

**Authors:** Min Ju Shon, Adam E. Cohen

**Affiliations:** Department of Chemistry and Chemical Biology and Department of Physics, Harvard University and Howard Hughes Medical Institute, Cambridge, MA 02138, USA

## Abstract

Proteins adhere to DNA at locations and with strengths that depend on the protein conformation, the underlying DNA sequence and the ionic content of the solution. A facile technique to probe the positions and strengths of protein-DNA binding would aid in understanding these important interactions. Here, we describe a ‘DNA pulley’ for position-resolved nano-mechanical measurements of protein-DNA interactions. A molecule of λ DNA is tethered by one end to a glass surface, and by the other end to a magnetic bead. The DNA is stretched horizontally by a magnet, and a nanoscale knife made of silicon nitride is manipulated to contact, bend and scan along the DNA. The mechanical profile of the DNA at the contact with the knife is probed via nanometer-precision optical tracking of the magnetic bead. This system enables detection of protein bumps on the DNA and localization of their binding sites. We study theoretically the technical requirements to detect mechanical heterogeneities in the DNA itself.

## INTRODUCTION

### Mechanics of protein-DNA interactions

Site-specific protein-DNA interactions are critical for DNA replication, packaging, transcription and repair. Bulk measurements via chromatin immunoprecipitation followed by sequencing (ChIP-seq) provide genome-wide information on binding sites, (1) but do not provide thermodynamic or kinetic information, nor probe single-molecule variations. Optical profiling techniques can follow the motion of proteins along single stretched strands of DNA (2), or along curtains comprised of many aligned molecules (3). These techniques require fluorescent labeling and encounter the challenges with photostability and spatial resolution common to all fluorescence measurements. High-resolution AFM can identify bound proteins (4) and putative kinks (5) in DNA, but these measurements might be confounded by interactions with the surface. Solid state nanopores have been used to detect the presence of proteins bound to DNA through occlusion of the nanopore by the protein (6), and to locate proteins on the DNA by controlling passage of the DNA through the pore with optical tweezers (7).

A clever recent technique used one DNA molecule held in a dual-beam optical trap to scan against another molecule stretched by magnetic tweezers (8,9). By using a flexible DNA strand as the probe, this technique experiences ambiguity in the exact contact location between the probe and target strands of DNA.

We developed a DNA pulley system (without a wheel) in which a nanofabricated knife scans along a magnetically stretched molecule of dsDNA. This approach uses a rigid and chemically well-defined surface as the probe, and uses a very simple optical setup. Single molecules of dsDNA can be probed along their sequence with nanometer precision for measurement times >1 h. The DNA pulley system gave clear signatures of proteins bound to the DNA, but did not detect increases in flexibility associated with single-stranded nicks. We model the effect of a perfectly flexible joint theoretically, and discuss the signal-to-noise requirements of detecting nicks, joints, and sequence-dependent changes in bending modulus.

## MATERIALS AND METHODS

### DNA pulley construct

Molecules of λ phage DNA (48.5 kb) were attached on one end to the exterior of a square glass capillary (1 mm I.D., Friedrich & Dimmock, BMC-1-15-50) via a digoxigenin anti-dig linker, and on the other end to a superparamagnetic bead (1 μm diameter, Life Technologies, MyOne^TM^ Streptavidin C1) via a biotin-streptavidin linker. This tethering protocol (details in Supplementary Data) was adapted from references (10–12). The purpose of the capillary was to allow the DNA to be stretched parallel to the focal plane of the microscope without concern about interactions between the bead and the coverslip. Movie S1 shows displacement of the tethered beads indicating stretching of the DNA in the presence of a magnetic field gradient generated by a permanent magnet.

At a stretching force of 1 pN, the WLC model predicts a displacement of 13.9 μm for single-tethered beads. For double-tethered beads, the greatest displacement occurs when the tension is shared equally between the strands, implying a displacement of 12.9 μm or less, depending upon the locations of the DNA attachment points. Beads displaced by less than the population mode were visually apparent and were not used in experiments.

### Silicon nitride knife fabrication

**Figure 1.**
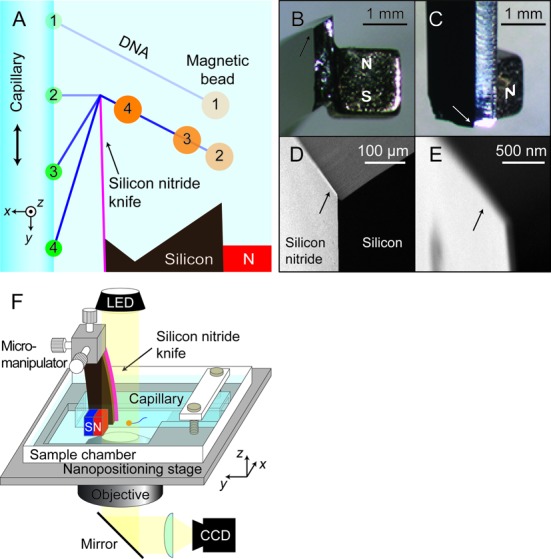
DNA pulley system. (**A**) Schematic of the experiment. The silicon nitride knife is held fixed, while a square capillary carrying a magnetically stretched molecule of DNA on its exterior is brought toward the knife edge (1) until the DNA makes contact with the blade (2). Further movement of the capillary drags the DNA over the blade edge in a pulley fashion (3,4). (**B**–**E**) Images of the blade and magnet. Arrows indicate location of the blade. (**B** and **C**) Stereomicroscope images of a silicon wafer supporting a silicon nitride blade (not visible) on one face and a permanent magnet glued to the opposite face. (**D­** and **E**) Scanning electron microscope images of the silicon nitride blade. The blade protrudes 10–20 μm from the silicon wafer and has a thickness of 55 nm. (**F**) Experimental setup. The capillary carrying the DNA is mounted rigidly in a sample chamber, which is held by a piezoelectric positioning stage. Coarse approach of the blade to the DNA is achieved via a micromanipulator; the blade is then held fixed while the capillary and sample chamber are moved by the piezoelectric stage.

A 55 nm film of low-stress silicon nitride was deposited on the clean (100) face of a silicon wafer via chemical vapor deposition (Supplementary Data). The film thickness was measured by ellipsometry. The wafer was manually cleaved into slivers 4 mm × 15 mm. A silicon nitride overhang (the blade) was formed by a selective Si etch (KOH, 30% w/v, 75°C, 20–30 min). The slivers were then carefully washed in distilled water and air dried. Examination of the edges under a dissecting microscope showed small free-standing films of silicon nitride protruding past the etched edges. Examination in a scanning electron microscope showed a smooth blade, free of cracks or grooves, with a thickness of 55 nm, an overhang length of typically 10–20 μm (Figure 1D–E), and a length along its edge of 1–2 mm. A NdFeB magnet (1/16’ cube, K&J Magnetics, B111) was glued to the back face of the silicon sliver to provide the stretching force.

### Measurement setup

The DNA-coated capillary and the nitride blade were mounted in a sample chamber as shown in Figure 1A and F. The capillary and magnetically stretched DNA both lay in the focal plane of an inverted microscope. The blade was aligned with its face in the *y-z* plane, parallel to the surface of the capillary (see Figure 1A and F for coordinate system), and its edge along the *z*-axis, perpendicular to the image plane of the microscope. A manual micromanipulator was used to position the blade at *x* ≈ 2 μm from the face of the capillary and *y* ≈ −2 μm from the strand of DNA. The tip of the blade was positioned at *z* ≈ −5 μm below the DNA to prevent the DNA from unhooking during scanning. Thereafter the blade was kept stationary and the capillary carrying the DNA was positioned via a piezoelectric nanopositioning stage (Mad City Labs, Nano-LP100). Scans were along the *y*-axis, typically 12–14 μm in length, at 1 μm/s. A transmitted-light image of the bead was recorded on a CCD camera and used to track the location of the bead with nanometer precision using a fast tracking algorithm (13). The entire apparatus was enclosed in a box to block air currents and thermal fluctuations, and was mounted on a vibration isolation table.

## RESULTS AND DISCUSSION

### Calibration of DNA pulley

**Figure 2.**
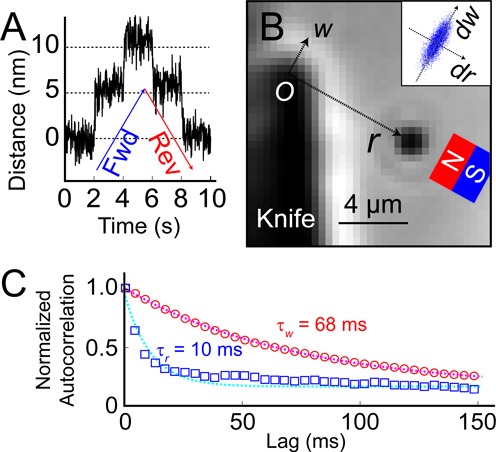
Calibration of the DNA pulley. (**A**) Localization of a bead immobilized on a coverslip. A piezo stage displaced the sample in 5 nm steps. (**B**) Coordinate system for describing bead motion in the DNA pulley. The knife blade is on the left, and a DNA-tethered bead is stretched from the point of contact with the blade toward the magnet (not visible). The origin, *O*, is at the tip of the knife, the *r*-axis is along the direction of magnetic force, and the *w-*axis is orthogonal to the *r-*axis. (Inset) Thermal fluctuations of the tethered bead. The fluctuations were smaller along the *r-*axis (stretching the DNA) than along the *w*-axis (rotation about *O*). (**C**) Normalized autocorrelation functions of the bead displacement. Thermal fluctuations along the *r*-axis decayed faster than along the *w*-axis, as expected for the stiffer spring constant along the *r*-axis. Autocorrelation functions were calculated from 12 000 measurements acquired at 200 Hz.

The DNA pulley system depends critically on the ability to track the motion of the bead with high precision. Jitter in the measured bead locations can arise from optical or electronic noise in the images; from drift or vibrations of the sample chamber; or from Brownian fluctuations of the bead itself. We characterized each contribution to the noise in turn. To quantify the contribution from measurement noise (optical and electronic) we simultaneously tracked two beads affixed to a dry coverslip. Measurement noise manifested as apparent fluctuations in the vector joining the beads. These fluctuations had an r.m.s. amplitude of 0.19 nm in a 1 Hz bandwidth, implying a single-bead tracking precision of 0.13 nm along each axis. The stage introduced an additional common-mode position noise of 2 nm along each axis, and had a minimum step size of 5 nm. The system had long-term thermal drift of ∼1 nm/min. A detailed analysis of the temporal structure of the mechanical drift is in Supplementary Figure S5. Steps in the piezo position of 5 nm were clearly resolved (Figure 2A).

When a bead was held by its DNA tether, its fluctuations in position were significantly larger than the measurement noise. The fluctuations formed an ellipse, with principal axes $\hat r$ parallel and $\hat w$ perpendicular to the direction of DNA stretching (Figure 2B). The standard deviations of the fluctuations were $\sigma _r$ = 32 nm and $\sigma _w$ = 89 nm. We calculated the autocorrelation function of the position fluctuations along each axis, and fit to an exponential decay, with decay times $\tau _r$ = 10 ms and $\tau _w$ = 68 ms (Figure 2C). These parameters set the fundamental limits on how precisely the bead's center of mass can be localized. If one integrates for a time *t*, the r.m.s. uncertainty in the center of mass is $\sigma _{{\rm meas}} = \sigma _{{\rm therm}} \sqrt {\tau /t}$, where $\sigma _{{\rm therm}}$ is the width of the Brownian distribution (equal $\sigma _r$ or $\sigma _w$).

We estimated the stretching tension in the DNA (in the absence of the blade) from the relaxation time of the thermal fluctuations (14). From the relaxation time and the estimated Stokes drag on the bead we inferred a spring constant (Supplementary Eq. 2). From the WLC force-extension curve (Supplementary Eq. 3) we then inferred a force (Supplementary Figure S7). In these calibrations, we accounted explicitly for blurring due to the finite exposure time of the camera (Supplementary Figure S6D). These estimates implied a mean tension of 1.0 pN, which stretched the DNA to 85% of its 16.2 μm contour length. An uncertainty of ±5 ms in the relaxation time led to an uncertainty of ±0.25 pN in the force. The discussion below does not depend on the precise value of the force.

During an experiment, the bead-magnet distance varied by (δR ∼ 10 μm) while the mean distance was R ∼ 1 mm. The force, *F*, on the bead was proportional to the gradient of the magnetic field. For a dipolar *F* ∝ 1/*R*^4^. Thus the fractional variation in the force on the bead was approximately 4 δR/R ∼ 4%. We did not include these variations in tension in our analysis. When the DNA slid smoothly over the pulley, the tension was uniform throughout the strand. When the motion stalled, the tension between the blade and attachment point could be greater or less than the magnetic tension, depending upon the direction of motion.

### Operation and geometry of the pulley

**Figure 3.**
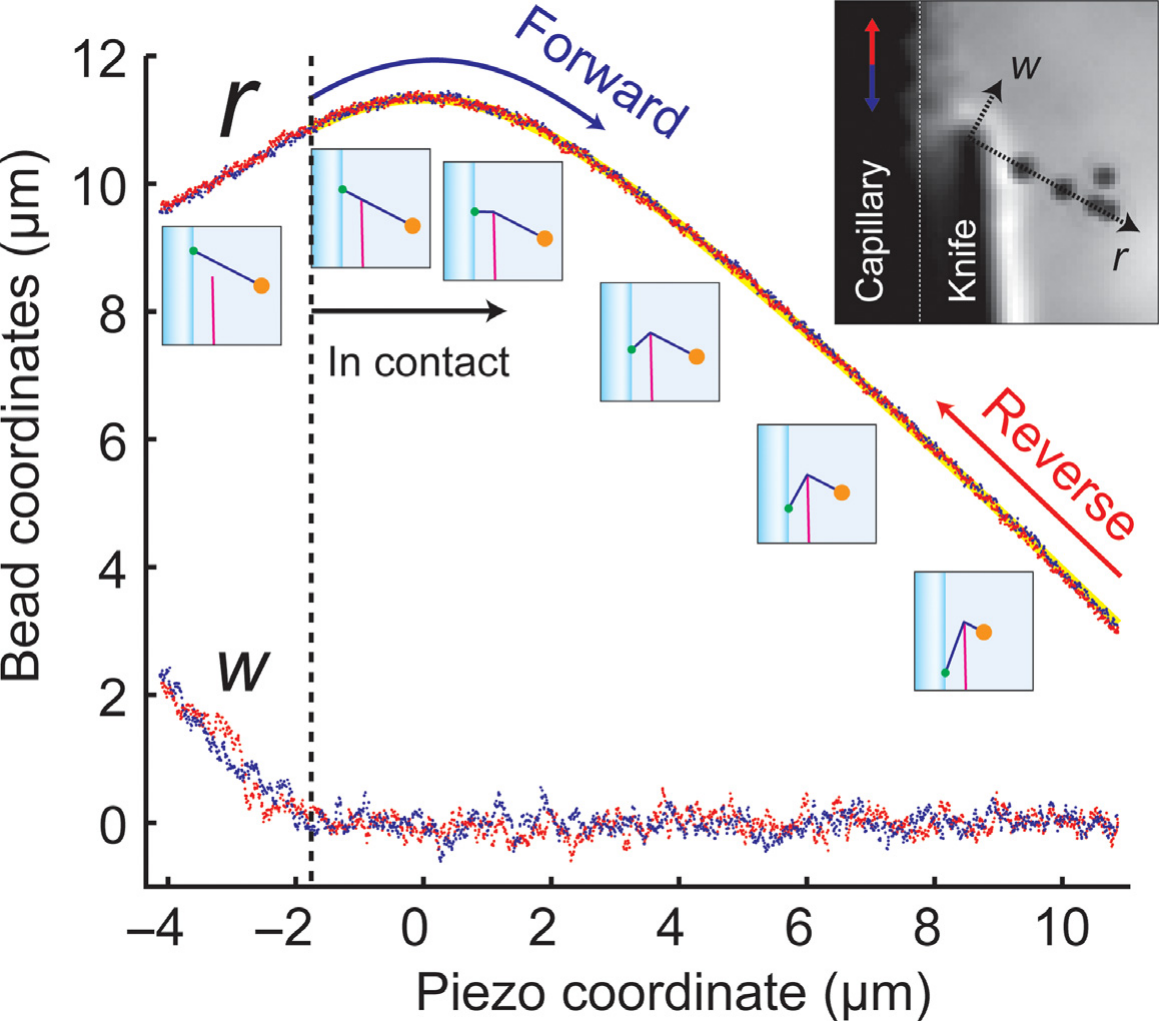
Bead motion in the DNA pulley. The piezo coordinate *p* is positive for downward motion of the capillary. For *P* < −2 mm, the DNA was out of contact with the blade. Initially the *w*- and *r*-coordinates varied with *P* according to their projection along the direction of piezo motion. For −2 mm < *P* < 0 mm, *r* increased as the DNA-capillary junction approached the blade. Thereafter the DNA-capillary junction receded from the blade and the bead was pulled toward the blade. Blue and red points represent measurements of bead location during a forward and reverse scan, respectively. Yellow line represents a fit to Equation (1).

Figure 3 shows a typical bead trajectory, Supplementary Movie S2 shows an animation, and Supplementary Movies S3 and S4 show videos of the scanning process. Before the DNA touched blade, the motion of the bead followed the motion of the capillary parallel to the *y*-axis. Following contact, the bead initially moved away from the blade along the *r*-axis and then moved toward the blade. The motion of the bead revealed the orientation of the *r-*axis, typically oriented at $\theta _{\bf F}$ ≈ 45° below the *x*-axis.

The bead trajectory can be predicted by solving the geometry of the pulley system. The parameters are the piezo movement along the *y*-axis, *P*, the separation of the blade from the capillary, *d*, and the total contour length of the DNA, $l_0$, assumed to remain constant throughout a scan. We define *P* = 0 when the line joining the surface attachment point and the knife edge is purely along the *x*-axis. The radial displacement of the bead, *r*, is given by:

(1) \begin{equation*} r = l_0 - \sqrt {p^2 + d^2 } .\end{equation*}

The transverse displacement, *w*, is zero as long as the DNA is in contact with the blade. Figure 3 shows a fit of this model to a scan trajectory. This simple model lets us map motion of the piezo scanner to motion of the DNA contour along the pulley. Deviations from Equation (1) indicate the presence of mechanical heterogeneities in the DNA.

### Protein bumps on DNA

We tested whether a protein, EcoRI, bound to the DNA introduced a detectable bump in the bead trajectories. EcoRI is a type II restriction endonuclease that cleaves its recognition sequence (5′-GAATTC-3′) with high specificity. In the presence of calcium ions, enzymatic cleavage is inhibited without loss of binding activity and specificity, as a result forming stable protein-DNA complexes on specific sites (15). λ-DNA contains five recognition sites for EcoRI spaced ∼2 μm from each other, making this protein a convenient source of sparse bumps.

**Figure 4.**
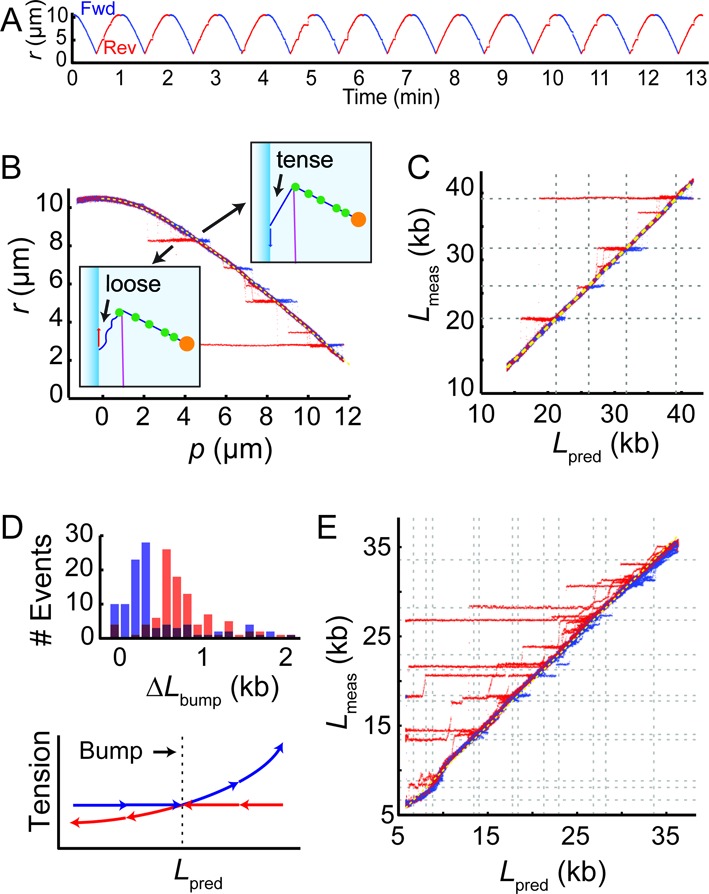
Protein bumps on the DNA pulley. (**A**) Scanning experiments with EcoRI-incubated pulleys revealed reproducible pausing of the bead at specific sites. (**B**) The data in (**A**) were collapsed and plotted as a function of piezo coordinate *p*. The cartoons explain the difference in the induced tension between the forward and reverse scan. (**C**) Mapping of the pause locations onto the EcoRI recognition sites (dashed lines). (**D**) Top: histogram of pause sizes for forward and reverse scans. Here Δ*L*_bump_ represents the absolute value of change in distance between the capillary-DNA junction and the DNA-blade junction during the interval in which the DNA is stuck (i.e. this would be the amount by which the DNA slid if it were sliding freely). Bottom: cartoon showing tension in the DNA segment between the capillary and the blade, before and after the EcoRI becomes hooked on the blade. In the forward scans, the tension increases after the EcoRI contacts the blade, while in the reverse scans the tension decreases. (**E**) Pauses in a molecule of λ-DNA incubated in EcoRV, mapped onto the EcoRV recognition sites (dashed lines).

When the pulley was incubated with EcoRI (50 nM) under non-cleaving conditions (1 mM Ca^2+^ in Tris-HCl buffer, Supplementary Table S2), the bead trajectory showed reproducible pauses at specific locations (Figure 4A and B). The pauses lasted for variable amounts of time before the bead jumped back to the regular trajectory. To test whether these pauses coincided with EcoRI recognition sites, we mapped the scan trajectory onto the underlying DNA sequence. Figure 4C shows the location along the DNA of the blade as inferred from the bead tracking, $L_{{\rm meas}}$, versus the location predicted from the motion of the stage $L_{{\rm pred}}$ (Supplementary Data). Indeed, deviations between the measured and predicted trajectories occurred predominantly when the predicted DNA-blade contact was an EcoRI recognition site.

We explored the region of an EcoRI binding site in greater detail. The blade was scanned repeatedly over a single binding site at 1 μm/s. Bumps in the trajectory were observed in 59/100 forward scans and 73/100 reverse scans (Supplementary Figure S8). The missing bumps likely indicate dissociation of bound protein. Free protein in solution could then re-bind to restore the bump. To estimate the spatial resolution of the DNA pulley system, we calculated the standard deviation of the measured bump location, presuming that the location of the true protein binding site was fixed relative to the DNA. Bump locations had a standard deviation of 85 nucleotides, corresponding to 25 nm.

Remarkably, the distribution of bump durations was very different between forward and reverse scans (Figure 4D, *top*). In the forward direction (bead moving toward blade), bumps were significantly briefer than in the reverse direction (bead moving toward magnet). We interpret this asymmetry by considering the tension in the DNA segment between the capillary and blade (Figure 4D, *bottom*). In the forward direction, pinning of an EcoRI molecule on the blade caused the tension to increase with further displacement of the capillary. In the reverse direction, pinning of an EcoRI molecule on the blade caused the tension to decrease. The shape of the waiting time distribution is quantitatively described by the ‘force ramp’ model of Evans and Ritchie (16), in which a labile bond is subjected to a tension that grows linearly with time.

We also incubated the pulley in EcoRV, another type II restriction enzyme. The recognition site of EcoRV (5′-GATATC-3′) is present 21 times in λ-DNA. The frequency of asperities increased drastically, with the locations roughly corresponding to the recognition sites. The molecular mapping of EcoRV recognition sites was less reliable than that of EcoRI, possibly due to nonspecific binding of EcoRV to non-canonical sites.

### Nanomechanics of single-stranded nicks

We next explored whether the DNA pulley could probe the endogenous mechanical properties of DNA itself. We asked whether there were variations in the DNA bending modulus along its length. Alterations to the canonical structure at the base-pair level (e.g. epigenetic modifications, mismatches or damage) modify protein binding and can affect the packaging or looping of DNA. Thus one would like to explore the sequence-dependent mechanical properties of DNA under a range of curvatures and in the presence of the many modifications found in Nature (17–19). These aspects of DNA mechanics have generated controversy in the literature, recently reviewed in detail in references (20,21). Variations in the bending modulus should affect the contour of the DNA around the knife edge, and thereby the displacement of the bead.

We studied the mechanics of a single nick in the phosphodiester backbone. The nick preserves base-stacking interactions, so it does not affect mean flexibility for thermally accessible curvatures (22,23). However, under strong bending a nick facilitates formation of a floppy joint, presumably via disruption of base stacking in the nicked strand (18). We used the enzyme Nb.BbvCI (New England Biolabs), to introduce single-stranded nicks at seven sites in λ-DNA. The pulley construct was then incubated in EcoRI under non-cleaving conditions. The molecules of EcoRI served as fiducial marks which helped locate the nick sites. Despite a thorough search at a precision of 25 nm (as determined from the repeatability of localizing EcoRI binding sites), we did not detect any mechanical signature of these nicks (Supplementary Figure S10).

**Figure 5.**
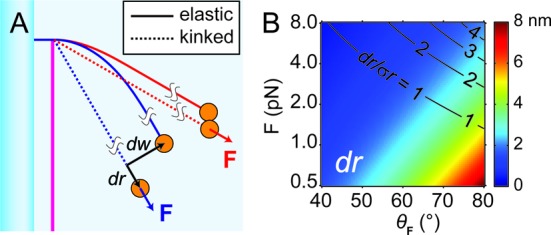
Comparison between kinking and elastica model of DNA bending. (**A**) Cartoon of the DNA pulley comparing the two conformations: elastic bending (solid curve) and kinking (dotted line). The blue and red sets of lines show that the impact of kinking on bead displacements, *dr* and *dw*, depends on the direction of the applied force vector, F. (**B**) Calculation of *dr* for experimentally feasible range of F. |F| is the magnitude of the force, and $\theta _{\bf F}$ is the angle between F and *x*-axis. The ability to resolve length changes *dr* depends on the ratio of *dr* to the amplitude of the thermal fluctuations along $\hat r$, *σ*_r_. Black contours indicate lines of constant *dr*/*σ*_r_.

To interpret this result, we calculated the expected bead displacement due to formation of a sharp kink at the blade (Figure 5A). We assumed a perfectly sharp blade (an admittedly crude approximation for a blade whose width is comparable to the persistence length). The mean contour of the smoothly bent DNA is described by a family of mathematical functions known as the elastica (24). In our case the boundary conditions are that the DNA far from the blade must be oriented along the magnetic force, and at the blade the DNA must be perpendicular to the blade edge. In the kinked state, we modeled the DNA as following a straight line from the blade toward the magnet.

Figure 5B shows the expected bead displacement along *r* as a function of the strength and direction of the magnetic force. The displacement is ∼2 nm for the 1 pN force and 45° angle range in our experiments. Such a displacement is, in principle, detectable with sufficient signal averaging. We attribute the absence of a detectable signal to the 55 nm width of the blade. The modest curvature around the blade tip may have been insufficient to disrupt base stacking and introduce a kink in the nicked strand. A sharper blade might increase the ability to resolve kinks and other small mechanical heterogeneities.

## CONCLUSION

The DNA pulley system provides a facile means to profile the locations of proteins bound to a molecule of DNA, without use of fluorescent labels or optical tweezers. The spatial resolution of the mechanical maps exceeds the diffraction limit. Here we used the DNA pulley to create a detailed mechanical map of the EcoRI binding site. This protocol could be readily adapted to identify previously unknown protein-DNA interaction sites. A key merit of the purely mechanical measurements is that they could be performed in complex media, such as cytoplasmic extracts, without concern for background autofluorescence and without need to fluorescently label putative DNA binding proteins. AFM imaging has been a powerful tool for localizing proteins bound to DNA (5,25–26), although the need for surface immobilization can raise concerns about surface artifacts. The DNA pulley combines spatial resolution of AFM with the ability of magnetic tweezers to apply controlled tension to the molecule.

To probe the endogenous mechanical variability in DNA, the pulley system will require a sharper blade. Extremely thin membranes of silicon nitride can be formed using advanced fabrication techniques. Diamond microtome blades also achieve nearly atomic sharpness. In principle, an appropriately supported graphene sheet could provide the ultimate in spatial resolution. One will need to determine experimentally the limits of applied tension and blade radius of curvature for which the DNA does not break. Finally, functionalization of these blades with DNA-binding proteins or with probe sequences of single-stranded DNA or RNA may provide a means to measure chemically specific interactions with DNA as a function of position along its contour.

## Supplementary Material

SUPPLEMENTARY DATA
