# Supplementary material for: Nano-mechanical measurements of protein-DNA interactions with a silicon nitride pulley
Source: Nucleic Acids Res. 2015 Sep 3;44(1):e7. doi: 10.1093/nar/gkv866 (PMC4705685; doi:10.1093/nar/gkv866)
Supplement: SUPPLEMENTARY DATA [file supp_44_1_e7__index.html]

Nano-mechanical measurements of protein-DNA interactions with a silicon nitride pulley — SUPPLEMENTARY DATA 

# Nano-mechanical measurements of protein-DNA interactions with a silicon nitride pulley

## SUPPLEMENTARY DATA

- SUPPLEMENTARY DATA
- SUPPLEMENTARY DATA
- SUPPLEMENTARY DATA
- SUPPLEMENTARY DATA
- SUPPLEMENTARY DATA
