# Supplementary material for: Nano-mechanical measurements of protein-DNA interactions with a silicon nitride pulley
Source: Nucleic Acids Res. 2015 Sep 3;44(1):e7. doi: 10.1093/nar/gkv866 (PMC4705685; doi:10.1093/nar/gkv866)
Supplement: SUPPLEMENTARY DATA [file supp_gkv866_nar-01584-met-g-2015-File007.pdf]

Supplementary Data

Nano-mechanical measurements on DNA with a silicon nitride pulley

Min Ju Shon[1] and Adam E. Cohen[1,*]

[1] Department of Chemistry and Chemical Biology and [*] Department of Physics, Harvard University, Cambridge, Massachusetts 02138, United States

[*]cohen@chemistry.harvard.edu

Contents

1. Fabrication of the silicon nitride knife 3
2. Preparation of DNA construct 4
3. Measurement setup 6
4. Localization of fixed beads 7
5. Calibration of magnetic force on the pulley 9
6. Localization of protein-DNA complexes 12
7. Converting bead displacements to molecular coordinates for molecular mapping 13
8. Measurement of DNA bending around a single-stranded nick 15
9. Simulation of DNA bending using theory of elastica 16
10. References 19

List of Figures

Figure S1. Fabrication of silicon nitride knife. 3

Figure S2. Synthesis of the DNA pulley. 4

Figure S3. Precision of bead localization. 7

Figure S4. Calibration of piezoelectric stage. 8

Figure S5. Long-term mechanical drift. 8

Figure S6. Thermal fluctuations of a tethered bead. 10

Figure S7. Calibration of force on the DNA pulley. 11

Figure S8. Reproducible detection of protein bumps on the DNA pulley. 12

Figure S9. Geometry of the DNA pulley. 13

Figure S10. Measurement of DNA bending around a single-stranded nick. 15

Figure S11. Elastica model for the bending of DNA. 16

Figure S12. Calculation of DNA bending comparing elastica and kinked DNA. 17

List of Tables

Table S1. Protocol for the synthesis of DNA pulley 5

Table S2. Composition of scanning buffer 6

1. Fabrication of the silicon nitride knife

Nanofabrication of the silicon nitride blade

55 nm of low-stress silicon nitride was deposited on a 400 μm-thick (100) silicon wafer using low-pressure chemical vapor deposition (Figure S1). The thickness of the nitride film was measured by ellipsometry. The nitride-coated wafer was hand-diced into 15 mm × 4 mm slivers, briefly rinsed with acetone, cleaned by air plasma for 10 min, and etched in 30% KOH at 75 °C for 20–30 min. A fresh blade was prepared before each experiment.

KOH etching along the straight (111) crystal plane is anisotropic and slow. However, we noticed that when the cleaved wafer had a somewhat curved edge, the etching proceeded much faster (~1 μm/min), possibly by bypassing (111) planes. The etch process left a 15–20 μm silicon nitride overhang. This procedure is robust down to membranes of thickness 30 nm. Below 30 nm, the membrane had a tendency to break upon drying due to surface tension forces.

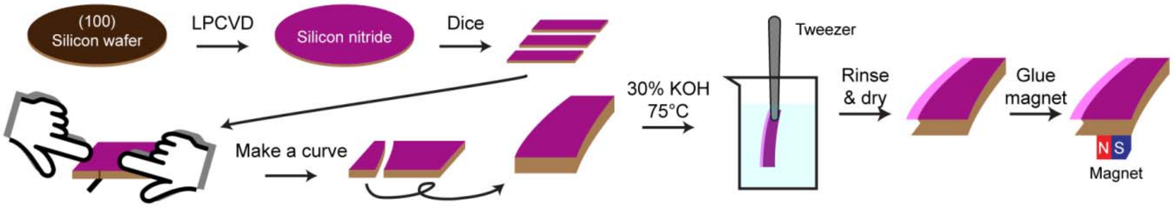

Figure S1. Fabrication of silicon nitride knife. Low-stress silicon nitride film was prepared on a silicon wafer by low-pressure chemical vapor deposition (LPCVD). Silicon under the nitride film was etched by 30% KOH, exposing the nitride blade. The curvature on the edges of the silicon sliver facilitated etching on the sides.

Attachment of magnet to the knife

A 1/16" cubic NdFeB magnet (K&J Magnetics, B111) was glued with epoxy on the back of the knife. The north pole of the magnet was oriented facing the front of the blade. The location of magnet determined the angle and strength of the magnetic force on the pulley, typically pulling the bead with ~1 pN of force at 45° relative to the flat surface of the blade.

2. Preparation of DNA construct

λ-DNA conjugation to bead and surface

λ-DNA (48.5 kb, 16 μm, New England Biolabs) was prepared following a published protocol (1) (Figure S2A) with slight modifications. Circular λ-DNA was dissolved at a concentration of 0.5 mg/mL (15.6 nM) in Tris-HCl buffer with 0.5 M NaCl. The molecule was linearized by heating to 90 °C for 10 min, followed by fast cooling in ice for 5 min. The linearized λ-DNA was then annealed with a 12-nt ssDNA oligo 5' labeled with digoxigenin (dig-12; Integrated DNA Technologies), and complementary to one terminal 12-nt ssDNA overhang (*cos* site). The dig-12 oligo was added at a concentration of 6 nM, selected to be the limiting reagent. The reaction was run for 1 h at room temperature. A second oligo, 5'-labeled with biotin, and complimentary to the other *cos* site, was then added at a concentration of 200 nM and incubated for 1 h at 4 °C. The hierarchy in relative concentrations enabled purification for doubly-labeled λ-DNA by anti-digoxigenin-coated capillaries.

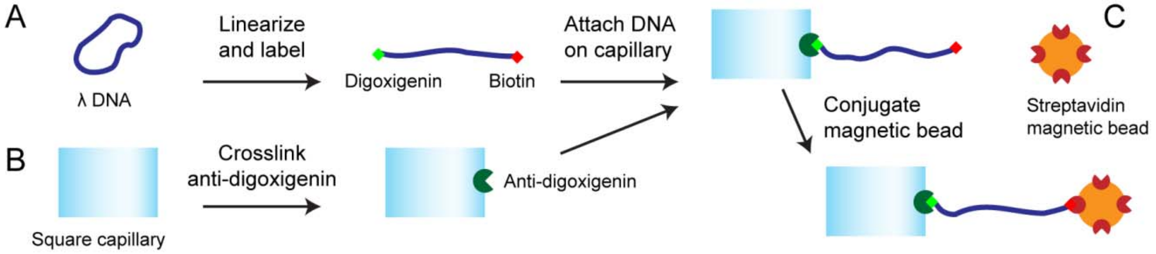

Figure S2. Synthesis of the DNA pulley. (A) A circular form of λ-DNA (blue strand) is linearized and labeled with digoxigenin (green square) and biotin (red square). (B) The exterior of a square capillary is coated with anti-digoxigenin. (C) The capillary with tethered λ-DNAs is labeled with a streptavidin magnetic bead.

Surface attachment of λ-DNA and labeling by magnetic beads

The flat outer surface of square glass capillaries (1 mm I.D., Friedrich & Dimmock, BMC-1-15-50) was used as a substrate for the DNA pulley. These capillaries provided a convenient means to orient the pulley constructs in the focal plane of the microscope. The attachment of λ-DNA (Figure S2B) was adapted from published protocols (2). The sequence of reactions is detailed in Table S1. The aldehyde modification and all subsequent reactions were carried out in PCR tubes (1 capillary per tube) at room temperature. Briefly, the glass surface was activated with glutaraldehyde, amino-modified with APTES, and coated with anti-digoxigenin. The anti-digoxigenin-coated capillary was mixed with labeled λ-DNA. Streptavidin-coated magnetic beads (Dynabeads® MyOne™ Streptavidin C1, Life Technologies, 1 μm diameter, washed following manufacturer's protocol) were then coupled to the DNA on the capillary (Figure S2C). Finally, ligase was added to repair the nicks in the construct (except when nicks were intentional-

ly introduced). Capillaries with attached beads were then transferred to the measurement chamber.

Table S1. Protocol for the synthesis of DNA pulley

Step	Reagent	Duration	Rinse
Capillary cleaning	Acetone<br>Alconox solution (sonication)<br>Air plasma after drying	1 min<br>10 min<br>5 min	N/A<br>Water<br>N/A
Amine modification[a]	(3-Aminopropyl)triethoxysilane (Sigma)	4 h	N/A
Aldehyde modification	0.5% Glutaraldehyde (Sigma) in PBS	30 min	PBS
Antibody crosslinking	20 μg/mL Anti-digoxigenin (Roche) in PBS	30 min	WB[b]
DNA tethering	200 pM Biotin- and dig-labeled λ-DNA in PBS	1 h	WB
Surface passivation	WB	15 min	N/A
Bead coupling	4 pM Streptavidin-coated magnetic beads[c] in BB[d]	1 h	WB
Ligation	20 units/μL T4 DNA ligase (New England Biolabs) in T4 DNA ligase reaction buffer	1 h	WB
Bead passivation	50 pM biotinylated oligonucleotides in BB	30 min	PBS
Storage	WB		N/A

[a] Amine modification was carried out in a glass desiccator under vacuum with vapor-phase silane.
[b] WB (wash buffer): 1× TAE buffer with 130 mM KCl, 4 mM $MgCl_2$, 20 μg/mL acetylated bovine serum albumin, 80 μg/mL heparin.
[c] Streptavidin-coated beads (Dynabeads® MyOne™ Streptavidin C1, Life Technologies) were rinsed before use following manufacturer's protocol.
[d] BB (binding buffer): WB with 1 M NaCl

3. Measurement setup

Sample chamber and stages

A capillary with DNA pulley was mounted in a custom Teflon/aluminum chamber with a glass bottom for imaging (Figure 1F in the main text). The chamber was loaded with 500 µL of sample solution and mounted on a nanopositioning piezo stage (Mad City Labs, Nano-LP100). The piezo stage was mounted on a manual micropositioning stage (Mad City Labs, MicroStage) for coarse positioning. The silicon nitride knife was clamped on a separate *xyz*-micromanipulator (Newport) and was aligned relative to the capillary. The knife-edge was aligned orthogonal to the *x*-*y* plane to avoid out-of-plane movements in the bead during the scanning.

The entire setup including the microscope was enclosed in a box to block air currents and supported on a vibration-isolation optical table. Shielding of air currents was essential to obtaining low drift: without the shield, drift was ~10 nm/min, and with the shield, drift was ~1 nm/min.

Optical setup

Measurements were performed on an inverted microscope (Olympus IX71). The sample was illuminated from above with a white LED. The image of the magnetic bead, silicon nitride knife, and capillary was collected with a 40× air objective (Olympus, N.A. 0.60), passed through a 3× beam expander, and recorded with an EMCCD camera (Andor DU-897-UV, 16-µm pixels), operated without electron-multiplying gain. Frame rates were selected between 2 and 200 Hz depending on the measurement.

Composition of sample buffer

For all DNA pulley experiments, we used the following Tris-HCl buffer. 1 mM of $CaCl_2$ was added for experiments with EcoRI/EcoRV.

Table S2. Composition of scanning buffer

Materials	Concentration
Tris-HCl (pH 8.0)	10 mM
NaCl	100 mM
Polyvinylpyrrolidone	0.1%
Tween-20	0.1%

4. Localization of fixed beads

Bead-to-bead distance measurements

To estimate the precision of bead localization we imaged two beads fixed on a glass coverslip. The beads were tightly fixed on the surface, so apparent fluctuations in bead-to-bead distance arose purely from measurement error. The position of the coverslip was advanced in 5 nm steps and imaged with an exposure time of 20 ms. Movies were averaged over 1 s and the bead coordinates were extracted by a radial symmetry-based algorithm on 15×15 pixels region of interest.(3)

Regardless of the underlying movement of the piezo stage, the measured distance between the two beads was nearly constant, showing tight correlation in the movements (Figure S3). The fluctuation in the inter-bead distance, σ_d, measured at 1 Hz was 1.9 Å. The measurement of inter-bead separation involves two measurements of position, each associated with the same measurement error, so the precision of localization for a single bead is $1.9/\sqrt{2} = 1.3$ Å in a 1 Hz bandwidth.

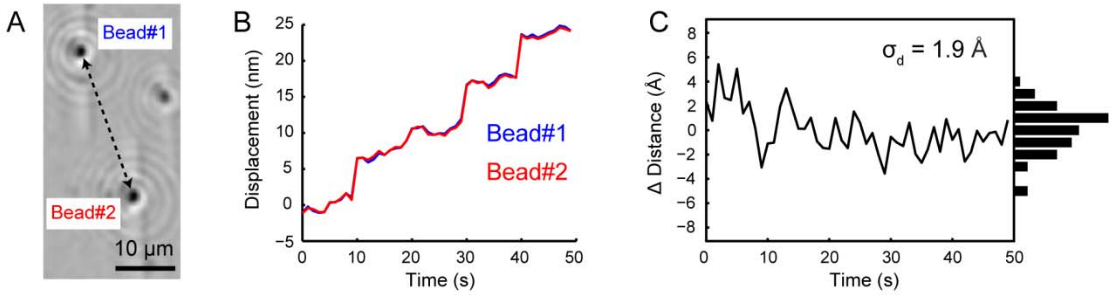

Figure S3. Precision of bead localization. (A) Two beads were fixed on a dry coverslip and imaged as a piexo stage moved the sample. The localization precision was inferred from the measurement error in the inter-bead distance (black dashed line). (B) Displacement of the two beads over time. The beads were moved by piezo stage with 5 nm steps. (C) Bead-to-bead distance over time.

Calibration of piezoelectric stage

The precision of the piezoelectric stage (Mad City Labs, Nano-LP100) was measured by following 5 nm steps over a total displacement of 1 µm. As shown in Figure S4, the motion of piezo stage introduced 2-nm r.m.s. mechanical noise in an 1 Hz bandwidth.

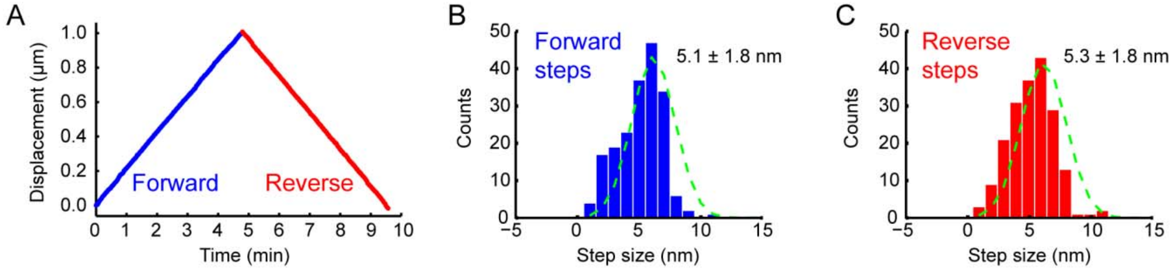

Figure S4. Calibration of piezoelectric stage. (A) Tracking 5 nm steps in a ramp of 1 μm total displacement. Forward (blue) and reverse (red) travel. (B–C) Distribution of step sizes for forward (B) and reverse (C) steps.

Long-term drift

The level of mechanical drift was checked by tracking a bead over many hours. As shown in Figure S5, the length-scale of mechanical drift was ~1 nm in 1 min, and ~10 nm in 1 h. Characterization of noise by Allan variance (4) showed a gradual increase of deviation over long time-scales due to the drift (Figure S5D).

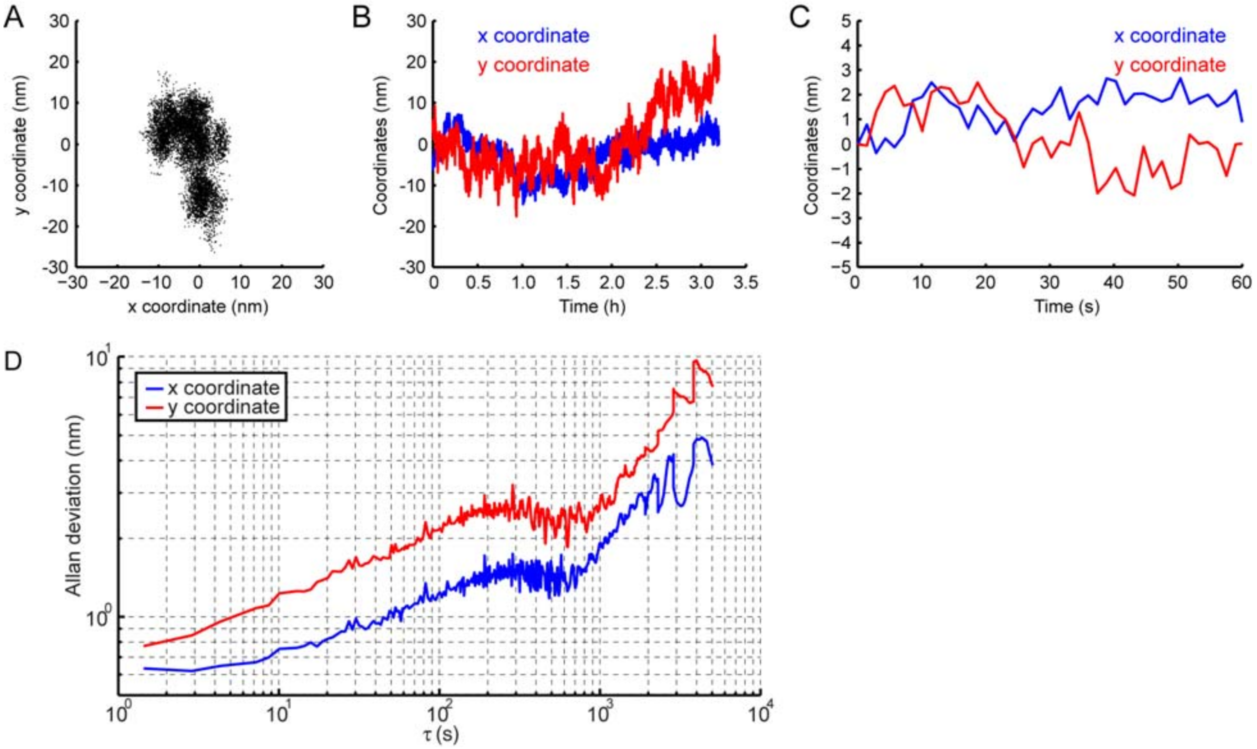

Figure S5. Long-term mechanical drift. Tracking of a nominally stationary bead at 1.4 s intervals for 3.2 hr. (A) 2-D distribution of measured bead locations. Each point represents one measurement. (B) *x*- and *y*-coordinates as a function of time. The low-frequency drift is ~10 nm/hr. (C) The drift on short time scale is ~1 nm/min. (D) Allan deviation of the x (blue) and y (red) coordinates for the data in (B).

5. Calibration of magnetic force on the pulley

Fluctuation measurement

A bead on a spring undergoes thermal fluctuations of mean square amplitude along each axis:

$$\sigma^2 = \frac{k_B T}{k} \tag{1}$$

where k is the spring constant, k_B is Boltzmann's constant and T is the absolute temperature. These fluctuations decay with a correlation time:

$$\tau = \frac{\gamma}{k} = \frac{6\pi\eta a}{k} \tag{2}$$

where γ is the drag constant, η is the dynamic viscosity of the medium, a is the radius of the bead.

The effective spring constants along the *r*- and *w*-axes are in turn related to the magnetic force. Along the *r*-axis, the force and extension are related by the modified Marko-Siggia formula for a WLC (5) :

$$F = \left(\frac{k_B T}{l_p}\right)\left[\frac{1}{4(1 - x/L_0)^2} - \frac{1}{4} + \frac{x}{L_0}\right] \tag{3}$$

where F is the force; x is the extension; l_p = 45 nm is the persistence length; L_0 = 16.2 µm is the contour length; and K_0 = 1000 pN is the elastic modulus (6). The effective spring constant is:

$$k_r = \left.\frac{dF}{dx}\right|_{x=r} \tag{4}$$

The magnetic force on the bead was measured from thermal fluctuation of the bead, *in situ* before scanning experiments (Figure S6). The bead fluctuation with all stages at rest and with the blade withdrawn was recorded at 200 Hz.

To quantify the bias induced by the finite exposure time of the camera, we ran a simple numerical simulation of a Brownian particle in a harmonic potential with a 10 ms relaxation time. We used 0.1 ms time-steps and produced a trajectory of 10^6 steps. We then simulated the effect of the finite exposure time of the camera by averaging the trajectory in 5 ms discrete intervals. The autocorrelation functions of the raw and discretely sampled trajectories are shown in Figure S6D. Discrete sampling led to an underestimate of the variance in particle position by 17%, but a negligible influence on the autocorrelation function at non-zero lag. For motion of a particle with a correlation time of 70 ms, discrete sampling with 5 ms integration times led to an underestimate of position variance of only 2.5%.

To fit the autocorrelation functions, we discarded the data point at zero lag (on account of the bias from finite camera exposure). We then fit the remaining data to a function of the form A exp(-t/τ) + B. The purpose of the constant offset, *B*, was to accommodate slow drift in the measured bead position, which led to a plateau in the autocorrelation function at timescales long com-

pared to the relaxation time. Typical time scales of fluctuations were $\tau_r \sim 10$ ms along *r*, and τ_w ~ 70 ms along *w* (Figure S6B and C).

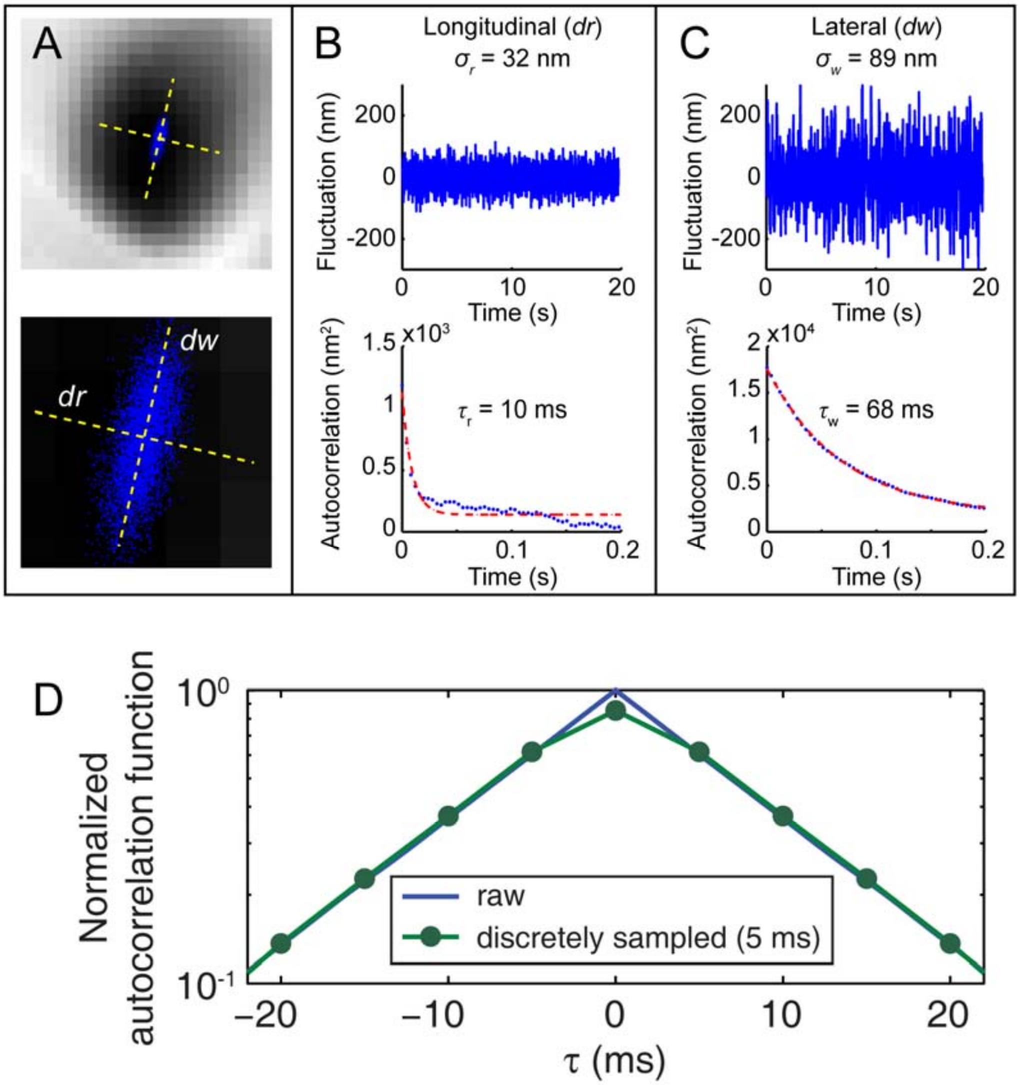

Figure S6. Thermal fluctuations of a tethered bead. (A) Image of fluctuating bead, recorded at 250 Hz and averaged for 1 min. Blue dots are the bead localization for each frame. Yellow dashed lines indicate the axes extracted from the principal axes of the ellipse of points. Bottom image is a close-up view of the distribution of points. (B) Fluctuation along the *r*-axis and autocorrelation of these fluctuations (bottom). (C) Fluctuations along the *w*-axis and autocorrelation of these fluctuations. (D) Effect of finite exposure time of camera on the autocorrelation function of Brownian trajectory. The autocorrelation function for the raw trajectory of simulated Brownian motion (blue without marker) was compared with that for the same trajectory after averaging and then discrete sampling with a 5 ms window.

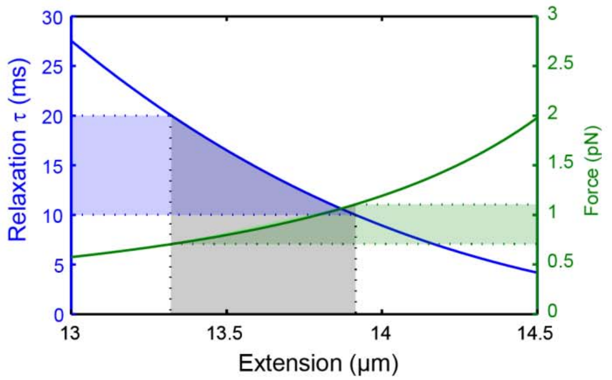

Figure S7. Calibration of force on the DNA pulley. (*Left y-axis*) Measured relaxation time of the bead fluctuations. (*Right y-axis*) Stretching force on the DNA. The relaxation time ranged from 10 to 20 ms. These values correspond to, by Eq. **(9)**, 13–14 μm extension of λ-DNA (85% extension) and ~1 pN of magnetic force.

6. Localization of protein-DNA complexes

Incubation with restriction enzymes

The DNA pulley was first incubated with 50 nM EcoRI-HF or EcoRV-HF (New England Biolabs, R3101 and R3195) in a scanning buffer (Table S2) containing 1 mM $CaCl_2$ (10 mM Tris-HCl, 100 mM NaCl, 0.1% polyvinylpyrrolidone, 0.1% Tween-20), for 1 h. After incubation, the number of pulley constructs on capillary was not reduced substantially, confirming that the catalytic activity of the restriction enzymes was lost.

The EcoRI-incubated DNA pulley constructs were studied by applying the same scanning condition as in the simple trajectory mapping. The scanning speed was 1 μm/s with a 100 Hz camera frame rate.

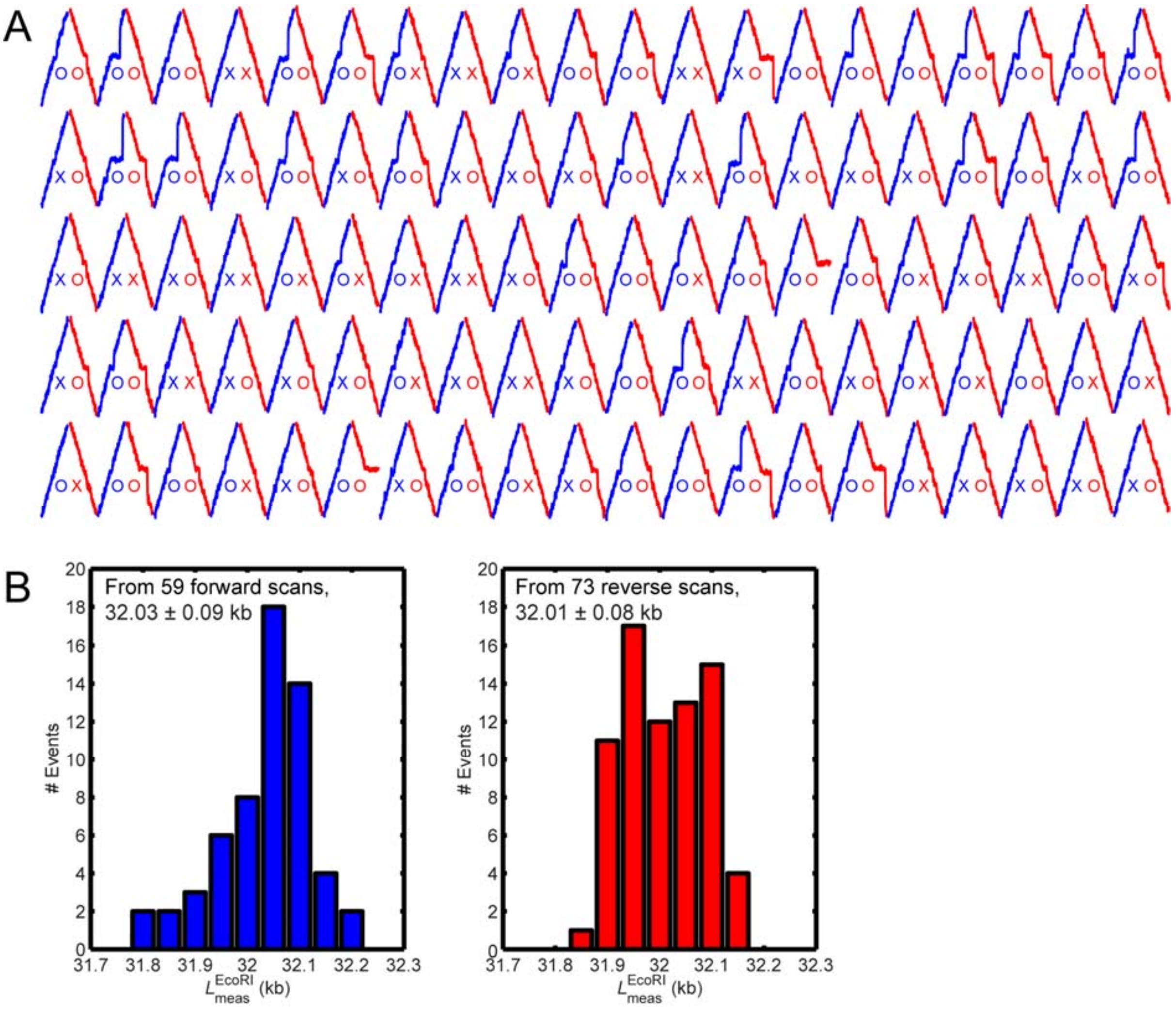

Figure S8. Repeated detection of protein bumps on the DNA pulley. (A) 100 consecutive forward (blue) and reverse (red) scans. EcoRI bumps were detected as deviations from the predicted bead trajectory. Bumps were detected in 59 forward scans and 73 reverse scans (denoted as "O"). (B) Histograms for the locations of EcoRI bumps detected in (A). The standard deviation in bump location in either direction was ~25 nm.

7. Converting bead displacements to molecular coordinates for molecular mapping

Geometry of the DNA pulley

We solved the geometry of the pulley system to parameterize the bead motion. We then mapped the measured bead coordinate, r, onto the location in the DNA sequence of the DNA-blade contact.

The analysis of the pulley geometry is greatly simplified if the blade edge is aligned parallel to the z-axis. Before experiments, the microscope focus was moved along the z-axis and the blade edge was aligned to be vertical. However, the angle of the magnetic force vector is not precisely controlled due to the need to manually position the magnet prior to gluing. Furthermore, the similar scale of the magnet size (1/16”) and the distance between the magnet and the bead implies that the magnetic field contour at the bead location is not simple to compute. The angle of the magnetic force in the x-y plane is trivially determined by noting the direction of DNA stretching; but the azimuthal angle, $\phi_\mathbf{F}$, relative to the z-axis must be included as a fitting parameter.

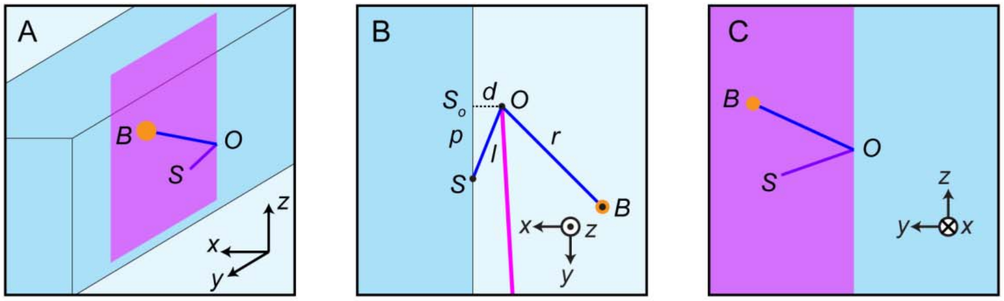

Figure S9. Geometry of the DNA pulley. (A) 3-D view of the pulley system. The points B, O, and S are the coordinates for the bead, pivot on the blade (origin), and surface attachment point, respectively. (B) The pulley viewed in the x-y plane, corresponding to the image plane of the microscope. (C) The pulley viewed in the y-z plane. Note that the three points B, O, and S do not lie in the x-y plane when $\phi_\mathbf{F} \neq \pi/2$, where $\phi_\mathbf{F}$ is the azimuthal angle of the force vector (measured relative to the z-axis). The apparent contour length observed in the microscope, l_0, is the projection onto the x-y plane of the true contour length L_0. Thus $l_0 = L_0 \sin(\phi_\mathbf{F})$.

When the z-component of the magnetic force vector is not zero, the bead is pulled out of the x-y plane, and therefore the z-coordinates of the three points B (bead), O (pivot or origin), and S (surface attachment) can be different (Figure S9). The focus of the microscope is adjusted so that only B is located in the image plane. Translation of the capillary (and hence S) along the y-axis does not change the z-coordinate of B, i.e. the bead remains in the focal plane during the scanning process, regardless of a vertical offset in the stretching force.

From Figure S9B, we can relate the observed bead coordinate, r, to the piezo coordinate, p. Let d be the distance between the capillary and the knife, and let S_0 be the position on the capillary closest to the knife edge. The DNA-capillary attachment is inferred to cross S_0 when the DNA appears maximally extended along r. This piezo coordinate is defined as $p = 0$.

From the right triangle ΔSS_0O, we get

$$l^2 = p^2 + d^2. \tag{5}$$

Therefore, the extension of the DNA between O and B, r, is given by

$$r = l_0 - l = l_0 - \sqrt{p^2 + d^2} \tag{6}$$

where l_0 is the total extension of DNA projected onto the x-y plane. Note that l_0 is always shorter than the total contour length L_0 (16.2 μm) because: (a) the DNA is not fully extended by the stretching force, and (b) the z-component of magnetic force might be nonzero, pulling the molecule slightly out of the x-y plane.

Ratiometric measurements of length and molecular coordinate

The relation of the observed contour length projected into the x-y plane, l_0, and the total extension in 3-D, L_0, is

$$l_0 = L_0 \sin \phi_{\mathbf{F}} \tag{7}$$

where $\phi_{\mathbf{F}}$ is the azimuthal angle of the magnetic force vector. In our measurements, the two variables, L_0 and $\sin \phi_{\mathbf{F}}$ do not have to be separated. Hence we regard l_0 as a single fitting parameter, acknowledging that we do not know L_0 and $\sin \phi_{\mathbf{F}}$ independently. The progression of the blade relative to the molecule in the image plane, l/l_0, is the same as the one in 3-D space, L/L_0. Therefore, knowledge of the ratio l/l_0 is sufficient to infer the absolute position, i.e., the sequence coordinate of the DNA-blade contact.

Deviations from pure pulley motion due to protein bumps on the DNA

When a DNA-bound protein catches on the blade edge, the measured location of the bead deviates from the predicted location, and therefore the point of contact between the DNA and blade as inferred from bead tracking deviates from the point of contact predicted from piezo motion. From Eq. (6), the predicted location of blade, $L_{\text{pred}}(p)$, is:

$$L_{\text{pred}}(p) = \left(\frac{\sqrt{p^2 + d^2}}{l_0}\right) \times (48.5\text{ kb}). \tag{8}$$

The blade position inferred from bead tracking is:

$$L_{\text{meas}}(p) = \left(\frac{l_0 - r}{l_0}\right) \times (48.5\text{ kb}) \tag{9}$$

In the absence of bumps along the DNA, the measured position of the blade $L_{\text{meas}}(p)$ will be the same as prediction, $L_{\text{pred}}(p)$. By comparing L_{meas} to L_{pred}, deviation from regular trajectory can be followed as a function of sequence.

8. Measurement of DNA bending around a single-stranded nick

The capillary with appended DNA and beads was incubated with Nb.BbvCI at 37 °C for 1 h for nicking. The capillary was then washed with WB (Table S2) to remove nicking enzymes. The sample was then incubated with EcoRI, as in Section 6.

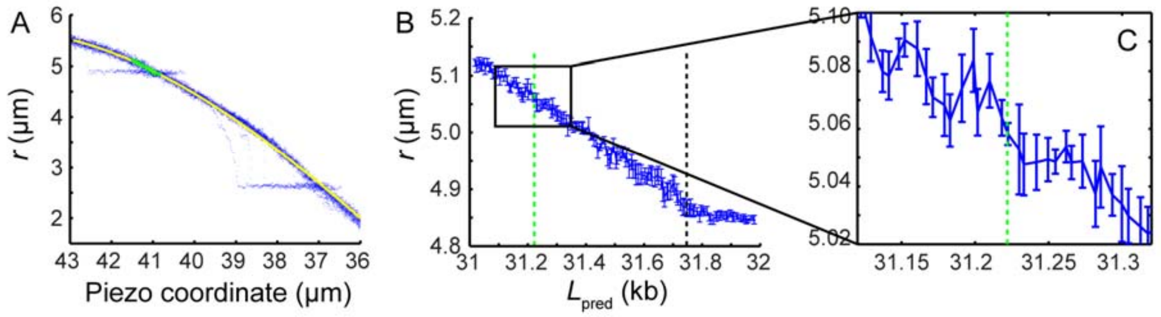

Figure S10. Measurement of DNA bending around a single-stranded nick. (A) Scans encompassing two EcoRI binding sites. The highlighted segment (upper left, green) includes an EcoRI site located close to a recognition site for the nicking enzyme Nb.BbvCI. (B) Slow scans around the highlited region in (A). Black and green lines indicate sites for EcoRI and Nb.BbvCI, respectively. Error bars indicate standard errors calculated from 7 measurements. (C) Close-up of (B) near the putative nicking site.

9. Simulation of DNA bending using theory of elastica

A sharp kink, i.e. a region of perfect flexibility, constitutes the largest mechanical signal one might hope to detect in DNA, and could arise e.g. from a single-stranded nick, a bubble, or possibly from covalent damage to the DNA. We thus sought to estimate the deviation in bead coordinate that would be induced by a running a point of perfect flexibility over the blade, assuming a perfectly sharp blade.

We compare the bead position under two scenarios: (1) the DNA bends smoothly around the edge of the (assumed perfectly sharp) blade, following the contour set by the applied tension and the continuum elasticity of the DNA; and (2) the DNA adopts a sharp kink at the blade and then extends in a straight line along the magnetic force. This calculation constitutes an upper bound on the signal. The blades used in our experiments were not sharp (55 nm diameter of curvature) compared to the persistence length, so our experiments would yield signals smaller than the estimates below.

For radii of curvature much smaller than the persistence length, the contour of a homogeneous linear elastic rod is described by a set of curves called the elastica. We modeled DNA as a simple elastic rod experiencing an external force on the ends. Due to the two-fold mirror symmetry of the force in the DNA pulley, we only consider the half with a magnetic bead.

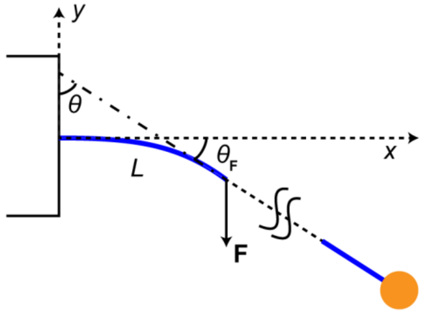

Figure S11. Elastica model for the bending of DNA.

The bending of DNA pivoted on the nitride blade can be thought of as a rod with one end clamped (Figure S11). Consider a segment of the tethered DNA with a length L, smaller than the persistence length (50 nm). The distal end of this DNA segment is pulled along the direction of magnetic tension. The angle between the tangent to the rod and the y-axis (θ) is related to the force and the rod length by (7):

$$L = \sqrt{\kappa/(2\mathrm{F})} \int_{\theta_0}^{\pi/2} \frac{cos\,\theta\, d\theta}{\sqrt{cos\,\theta_0 - cos\,\theta}} \qquad (10)$$

where κ is the bending modulus and $\mathrm{F} = |\mathbf{F}|$ is the applied force. The direction of magnetic force sets a boundary condition for the slope of curve. We solve for an elastica that asymptotes to the force angle ($\theta_0 = \pi/2 - \theta_\mathbf{F}$). Substituting $\theta_\mathbf{F}$ into the Eq. (10) gives a length scale, L^*, over

which the DNA transitions from a bent to a linear shape. The complete shape of the elastica regime (Figure S12, solid curves) is parametrically given by $\theta_{\mathbf{F}}$:

$$x = \sqrt{2\kappa/\mathrm{F}}\left[\sqrt{\cos\theta_{\mathbf{F}}} - \sqrt{\cos\theta_{\mathbf{F}} - \cos\theta}\right],$$
$$y = \sqrt{\kappa/(2\mathrm{F})}\int_{\theta}^{\pi/2} \frac{\cos\theta\, d\theta}{\sqrt{\cos\theta_{\mathbf{F}} - \cos\theta}}. \qquad (11)$$

As depicted in Figure S12A and B, the signal we are looking for is the difference in the bead position, resulting from a change in the local structure. If the DNA behaves as a perfect WLC, it will follow the solid curves. If the curvature introduced in the DNA is relieved by a localized kink, it follows a free-joint curve shown in dashed lines. To estimate the signal, the coordinates of the end of the DNA segment are compared for the two models.

A large force angle or a weak force is expected to give a large difference between the smoothly bent and the kinked state. The amplitude of the lateral displacement, *dw* is generally larger than the radial displacement, *dr* (Figure S12C and D).

One must compare the amplitudes of the displacements to the amplitudes and timescales of the thermal fluctuations in bead position. Thermal fluctuations are larger and slower for a weak force than for a strong force; and are larger and slower for measuring *dw* than for measuring *dr*. To detect kinks or other intrinsic mechanical heterogeneities in DNA will require (a) a sharper blade than we used, and (b) better long-term stability of the apparatus to permit averaging the bead coordinate over many relaxation time constants to achieve better tracking accuracy.

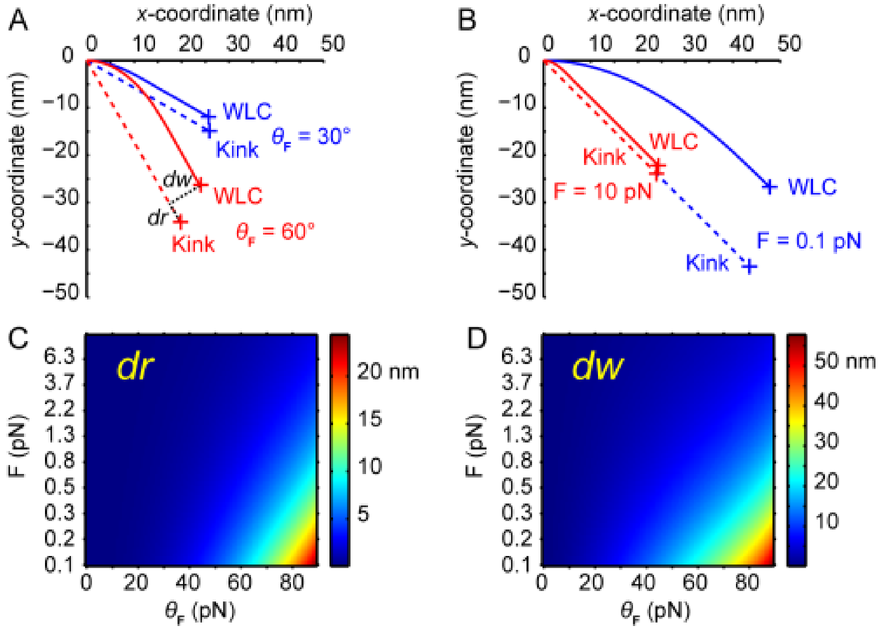

Figure S12. Calculation of DNA bending comparing elastica and kinked DNA. DNA is modeled as a rod with a persistence length 46.5 nm (solid curves) and compared to the kinked model (dashed lines). Crosses on the curves are marked keeping the contour lengths the same for the two cases. (A) Varying the force angle, $\theta_{\mathbf{F}}$, at a constant force magnitude F = 1 pN. The difference between the elastic and kinked model is greater at a larger angle. The longitudinal and lateral difference is designated as *dr* and *dw*,

respectively. (B) Varying the force magnitude, F, at a constant force angle $\theta_{\mathbf{F}}$ = 45°. The difference between the elastic and kinked model is greater at a smaller force. (C–D) Extended simulations of the plots in (A) and (B).

10. References

1. Zhou,R., Schlierf,M. and Ha,T. (2010) Chapter sixteen-Force–Fluorescence spectroscopy at the single-molecule level. *Meth. Enzymol.,* **475,** 405-426.

2. Han,L., Garcia,H.G., Blumberg,S., Towles,K.B., Beausang,J.F., Nelson,P.C. and Phillips,R. (2009) Concentration and length dependence of DNA looping in transcriptional regulation. *PLoS One,* **4,** e5621.

3. Parthasarathy,R. (2012) Rapid, accurate particle tracking by calculation of radial symmetry centers. *Nat. Methods,* **9,** 724-726.

4. Czerwinski,F., Richardson,A.C. and Oddershede,L.B. (2009) Quantifying noise in optical tweezers by allan variance. *Optics Express,* **17,** 13255-13269.

5. Marko,J.F. and Siggia,E.D. (1995) Stretching DNA. *Macromolecules,* **28,** 8759-8770.

6. Wang,M.D., Yin,H., Landick,R., Gelles,J. and Block,S.M. (1997) Stretching DNA with optical tweezers. *Biophys. J.,* **72,** 1335-1346.

7. Landau,L. and Lifshitz,E. (1986) Theory of Elasticity. Pergamon Press, Oxford, UK.
